# Arginine Methylation of hnRNP A2 Does Not Directly Govern Its Subcellular Localization

**DOI:** 10.1371/journal.pone.0075669

**Published:** 2013-09-30

**Authors:** Lexie R. Friend, Michael J. Landsberg, Amanda S. Nouwens, Ying Wei, Joseph A. Rothnagel, Ross Smith

**Affiliations:** 1 School of Chemistry and Molecular Biosciences, The University of Queensland, St. Lucia, Queensland, Australia; 2 Institute for Molecular Bioscience, The University of Queensland, St. Lucia, Queensland, Australia; Peking University Health Science Center, China

## Abstract

The hnRNP A/B paralogs A1, A2/B1 and A3 are key components of the nuclear 40S hnRNP core particles. Despite a high degree of sequence similarity, increasing evidence suggests they perform additional, functionally distinct roles in RNA metabolism. Here we identify and study the functional consequences of differential post-translational modification of hnRNPs A1, A2 and A3. We show that while arginine residues in the RGG box domain of hnRNP A1 and A3 are almost exhaustively, asymmetrically dimethylated, hnRNP A2 is dimethylated at only a single residue (Arg-254) and this modification is conserved across cell types. It has been suggested that arginine methylation regulates the nucleocytoplasmic distribution of hnRNP A/B proteins. However, we show that transfected cells expressing an A2^R254A^ point mutant exhibit no difference in subcellular localization. Similarly, immunostaining and mass spectrometry of endogenous hnRNP A2 in transformed cells reveals a naturally-occurring pool of unmethylated protein but an exclusively nuclear pattern of localization. Our results suggest an alternative role for post-translational arginine methylation of hnRNPs and offer further evidence that the hnRNP A/B paralogs are not functionally redundant.

## Introduction

In eukaryotic cell nuclei, nascent pre-mRNA transcripts (hnRNA) are packaged into ribonucleoprotein (RNP) complexes by a group of highly conserved, abundant proteins, the heterogeneous nuclear ribonucleoproteins (hnRNPs) A/B. These complexes, visualized on electron micrographs of non-nucleolar transcription units, appear as repeating globular RNP structures approximately 250 Å in diameter [1]. The hnRNP A/B proteins were isolated from cell nuclei in the form of RNA-protein particles sedimenting at around 40S [2] and later were found to package around 500–700 nucleotides of newly transcribed RNA [3], [4] The RNP particle arrangement on nascent hnRNA is nonrandom and sequence-dependent [1], [3], [5] and serves to condense and stabilize the transcripts and minimize tangling and knotting: this is especially relevant for long tracts of unspliced pre-mRNA [3], [6], [7]. Packaging also serves to shield the pre-mRNA from ribonucleases [3]. The pre-mRNA transcripts are not fully coated with hnRNP particles [1], [3], thus sequences essential for recognition and the subsequent removal of introns or for alternative splicing events, remain accessible. Despite some progress made in determining their positioning and assembly properties during transcript packaging [4], [7], [8] the mechanism(s) by which this group of proteins is selected for, or excluded from nascent transcripts within the nuclear milieu, has not yet been established.

The hnRNP paralogs A1, A2/B1 and A3 (hnRNPs A/B) share a high degree of sequence similarity with alternative splicing giving rise to multiple isoforms with diverse roles [9], [10]. Their modular structure consists of two tandem, N-terminal RNA recognition motifs (RRM) and a C-terminal glycine-rich domain (GRD) comprising several quasi repeats of arginine and glycine (in the RGG box) [11]. The RRMs for A1 are more similar in sequence to A3 than to A2/B1, whilst the converse is true for the GRDs [12]. The RGG box, proposed as an RNA binding motif and predictor of RNA binding activity, has been shown to modulate binding to single-stranded nucleic acids [13], [14], [15], [16], [17] and has been implicated in the nuclear import/export of certain hnRNP A/B isoforms [18], [19], [20]. There is also evidence that the GRD mediates self-association between the hnRNP A/B paralogs [21].

Arginine methylation is a major post-translational modification found in nuclear proteins that is catalyzed by a family of protein arginine methyl transferases (PRMTs) (reviewed in [17]). Of these, PRMT1 catalyses the sequential addition of two methyl groups to a guanidino nitrogen of arginine, forming asymmetric (*N* ^G^,*N* ^G^)-dimethylarginine (aDMA). This modification has no effect on net charge of the arginine side chain but does increase the steric constraints between the side chain and the RNA and thus influences the arginine-RNA association [17], [22], [23]. Most (65%) of the *N* ^G^,*N* ^G^-dimethylarginine found in the nucleus is in the hnRNP proteins that are complexed with hnRNA however, only 12% of the total arginines in these proteins are methylated, some more extensively than others [24].

The question as to why some of the hnRNPs are more extensively methylated on arginine residues whereas others are not remains unanswered. Furthermore, the functional consequences of arginine methylation remain ambiguous. One study has reported that inhibition of PMRT1 results in cytoplasmic accumulation of mammalian hnRNP A2 [19], suggesting that methylation regulates subcellular localization of A2, although a contrary observation was seen for the yeast homolog [20]. Another study has shown that methylation of A1 results in reduced affinity for single-stranded nucleic acids suggesting that this modification allows regulation of the interactions between hnRNPs and their target RNAs [14]. Given that the GRD (and the RGG box) of hnRNP A/B proteins has itself been suggested to influence the nucleocytoplasmic distribution of hnRNPs and implicated in hnRNP oligomerization and RNA binding, the question as to which of these functions methylation of arginine plays a role is an important one.

We recently reported that the hnRNP A/B paralogs display distinct and differential nuclear localization patterns that are dependent on RNA integrity and active transcription during the cell cycle [25]. Moreover, we found that the minor A/B splice variants A2b and B1b are predominantly responsible for cytoplasmic RNA trafficking whereas A2 and B1 are implicated in nuclear roles [10]. In these studies, the influence of methylation status was not investigated. Here we have investigated methylation of the hnRNP A/B family and show that the pattern of arginine methylation of A2 and B1 is markedly different from that of A1 and A3. Our data highlight distinct complementary patterns of +/− dimethylation of the arginine residues within a conserved RNA-binding motif that lies in close proximity to the RRMs. Significantly, we could not find any evidence that methylation of A2/B1 regulates nucleocytoplasmic localization or shuttling but instead suggest that methylation of key arginines within the RGG box may allow A/B paralogs to regulate their binding affinities in order to discriminate between different nascent transcripts.

## Materials and Methods

### Ethics Statement

All work involving animals was carried in accordance with guidelines approved by the University of Queensland Animal Ethics Committees (ethics approval number: 441/08). Wistar rats were sacrificed at 21 d by CO_2_ asphyxiation.

### Protein Purification

Native rat brain hnRNP A2 from 21 d Wistar rats and recombinant hnRNP A2 expressed in *E. coli* were isolated and purified as described previously [26], [27]. The pulldown procedure used to purify rat brain protein [28] was then adapted to isolate hnRNP A2 from HeLa [25], B104 [10] and SH-SY5Y [10] cultured cells. Cells previously grown to confluency and stored at −80°C were quickly thawed on ice and incubated for 5 min with 300 µL of lysis buffer (20 mM HEPES pH 7.4, 0.65 M KCl, 2 mM EGTA, 1 mM MgCl2, 2 M glycerol, 14 mM 2-mercaptoethanol, 0.5% IGEPAL Ca-630, 12 mM deoxycholic acid, 1 mM PMSF, Sigma protease inhibitor cocktail). Cells were scraped, repeatedly syringed through a 27-gauge needle and the resulting lysate centrifuged for 30 min. In a standard 1 mL pulldown assay, 100 µL of 100 mg/mL heparin and 200 µL of a 5×binding buffer (10 mM HEPES pH 7.5, 3 mM MgCl2, 40 mM NaCl, and 5% glycerol) were added to 0.5 mg of magnetic particles which had been labeled with either 500 pmol of A2RE11 or Telo4. Then, 700 µL of cell lysate was added and the mixture incubated on rollers for 2 h at 4°C. The buffered cell lysate and heparin were removed from the magnetic particles subjected to the first round of binding. A fresh aliquot of 700 µL of cell lysate, 100 µL heparin and 200 µL of a 5× binding buffer was added to the protein-bound particles and incubated on rollers for a further 20–24 h at 4°C. The magnetic particles were then washed and the bound proteins eluted in 50 µL of 30% ACN/0.1% TFA as previously described.

### Western Blotting

A2RE11 and Telo4 pulldown proteins separated by 12% SDS-PAGE were transferred to Immobilon-Fl membranes (Millipore, Billerica, MA) for Western blotting. Membranes were blocked for 1 h at room temperature with PBS containing 3% fish skin gelatin and then incubated for 1 h at room temperature with rabbit polyclonal anti-A2/B1 and anti-A3N [12], which recognize the alternatively spliced isoforms of hnRNP A2 and A3, respectively. After 3 washes with PBS/0.1% Tween-20, the membrane was incubated with Alexa 680-conjugated anti-rabbit IgG (Molecular Probes). Washed membranes were scanned at 700 nm using an Odyssey Infrared System (LI-COR Biosciences).

### Enzymatic Digestions

For native rat brain, endoproteinase digestions were performed either in 0.5 mL tubes (for LC-MS) or in-gel (MALDI-TOF, TOF/TOF and LC-MS/MS). For the former, sequencing-grade porcine trypsin (Promega) was reconstituted and digests were carried out according to the manufacturer’s instructions at an enzyme-to-protein weight ratio of 1∶50. In-gel digests were based on the procedure of Speicher and co-workers [29]. A digestion buffer of 40 mM ammonium bicarbonate, 10% ACN (pH 8.1) was used for the endoproteinase AspN (Sigma) and trypsin digests, or 25 mM Tris, 1 mM EDTA, 10% ACN (pH 8.5) for the endoproteinase Lys-C (Roche) digests, using 1 µg of the respective enzyme. For trypsin and chymotrypsin digestions prior to MS/MS analysis, a digestion buffer of 50 mM ammonium bicarbonate was used and 80 ng of enzyme was added prior to overnight digestion at 37°C. For thermolysin/AspN (Sigma) double digestions, gel pieces were rehydrated with 0.5 µg thermolysin in 50 mM ammonium bicarbonate for 4 h at 25°C, followed by addition of a further 0.5 µg thermolysin for 2 h at 60°C, and finally addition of 20 ng AspN overnight at 37°C. Extracted peptides were concentrated in a vacuum centrifuge and redissolved in 50% ACN/0.1% TFA (for MALDI-TOF) or 5% ACN/0.1% formic acid (for ESI-QTOF).

### LC-ESI-QTOF MS

Peptides resulting from trypsin digestion were separated by C3 reverse-phase HPLC using a gradient of 0–80% B (Buffer A = 0.1% formic acid, Buffer B = 90% ACN/10% Buffer A) at a flow rate of 300 µL/min. 10% of the eluate was analyzed on a QSTAR Pulsar ESI-QTOF (Applied Biosystems, Framingham, NH) operated in positive ion mode and equipped with a standard ionspray source. Data were collected over the range m/z 400–2000, processed and analysed using the Analyst QS software (Applied Biosystems).

For LC-MS/MS, endoproteinase digests were separated by C18 reverse-phase HPLC using a gradient profile of 0% B (0–5 min), then 0–60% B (5–65 min), then 60–90% B (65–67 min) and a flow rate of 10 µL/min. The eluate was analysed directly and data were collected over the range m/z 400–1600 for 1 s, followed by information dependent acquisition of the four most intense ions for 2 s and then dynamic exclusion for 180 s. Peak lists were generated and searched against the MASCOT LudwigNR database via the Australian Proteome Computation Facility to confirm protein identities and identify modified peptides. Taxonomy was restricted and methionine oxidation and arginine methylation were set as variable modifications, allowing for a mass tolerance of 0.5 Da (MS) and 0.2 Da (MS/MS) and up to two missed cleavages.

Higher quality sequence information was subsequently obtained for precursor ions corresponding to dimethylated peptides using a spray voltage of 4 kV with collision energy adjusted as needed. Data were then manually interpreted and compared to a theoretical fragmentation pattern of b- and y-ions and NH_3_ and H_2_O neutral losses.

### MALDI-TOF MS

Samples were mixed 1∶1 with α-cyano-4-hydroxycinnamic acid (CHCA) matrix (10 mg/mL in 50% ACN/0.1% TFA, Sigma) and spotted onto target plates. MALDI-TOF analysis was then performed on a Voyager-DE STR MALDI-TOF mass spectrometer (Applied Biosystems) operating in reflectron mode at an accelerating voltage of 20 kV. Spectra were typically acquired from a minimum of 500 shots, initially over a wide m/z range that was then narrowed according to the ions present in the sample.

TOF/TOF analyses were performed on a Bruker Autoflex MALDI-TOF/TOF at an accelerating voltage of 19 kV. Peptides of interest, selected using the LIFT feature on the instrument, were fragmented by collision-induced dissociation. Spectra were acquired using a minimum of 1000 shots and interpreted manually to determine peptide sequence. Spectra were also interrogated for diagnostic ions and neutral losses derived from arginine and dimethylarginine as described by Gehrig and colleagues [30].

### Edman Sequencing

Thermolysin/AspN double-digested A2 protein was separated on a reverse-phase C18 column as above. The eluate was split, with 10% analysed by ESI-QTOF and re-analysed by MALDI MS to identify the fraction containing the F244-Y275 peptide (m/z = 2829). The remaining 90% was collected manually and used for subsequent Edman sequencing of this peptide, performed on a ProcisecLC sequencer (Applied Biosystems, Foster City, CA) using *N* ^G^-methyl-L-arginine (MMA; Sigma,St Louis, MI), *N* ^G^,*N* ^G^-dimethyl-L-arginine hydrochloride (aDMA; Sigma), and *N* ^G^,*N* ´^G^-dimethyl-L-arginine dihydrochloride (sDMA; Calbiochem, La Jolla, CA) as calibrants in addition to standard amino acids.

### Cell Culture and Transient Transfection

Cultured cells were maintained at 37°C in a humidified 5% CO_2_ atmosphere in either DMEM/F12 (Sigma), 2 mM Glutamax (Gibco) plus 5% newborn calf serum (B104), DMEM/F12, 2 mM Glutamax plus 10% fetal bovine serum (SH-SY5Y) or DMEM (Gibco) supplemented with 10% newborn calf serum, 100 U/mL penicillin, 100 mg/mL streptomycin and 10 mM HEPES, pH 7.4 (HeLa). For transient transfections, cells grown to ∼50% confluency were transfected on either plain (HeLa) or fibronectin-coated coverslips (5 µg/50 µL; B104, SH-SY5Y) with plasmids expressing either A2-GFP, A2^R254A^-GFP or pEGFP-N1 control. 200 ng of plasmid DNA and 0.5 µL LF2000 was used per well. 16 h post transfection, cells were washed, fixed with 4% PFA in PBS, permeabilised and stained with Hoechst dye as previously described [25].

To analyze the endogenous protein distribution, cells grown for 16 h on coated coverslips were immunostained with a primary rabbit polyclonal antibody directed against hnRNP A2/B1 [12] at 1∶400 followed by a secondary Alexa 488 goat anti-rabbit antibody at 1∶4000 prior to analysis by confocal laser-scanning microscopy (LSM 510 Meta, Zeiss Inc., Oberkochen, Germany).

## Results

### Post-translational Modification of the hnRNP A/B Proteins

Previous experiments have shown that several of the RGG motifs in HeLa cell hnRNP A1 are dimethylated [14], [31]. Our aim in the current studies was to establish whether hnRNPs A2/B1 (hereafter referred to as A2, reflecting the predominant isoform) and A3, are likewise methylated and to determine the location of any post-translationally modified residues. From LC-MS experiments on a tandem quadrupole-TOF spectrometer we had earlier obtained the average mass of the major alternatively spliced isoform of A2 [12] and found that this mass exceeded that anticipated based on the primary amino acid sequence. The average mass of hnRNP A2 was 36,076±5 Da, 70±5 Da above the theoretical 36,006 Da average mass, making it likely that hnRNP A2 is post-translationally modified. Similarly, for hnRNP A3 we observed a mass discrepancy of 211 Da [12], which exceeds the expected mass based on amino acid sequence alone by a significantly greater amount.

**Figure 1:**
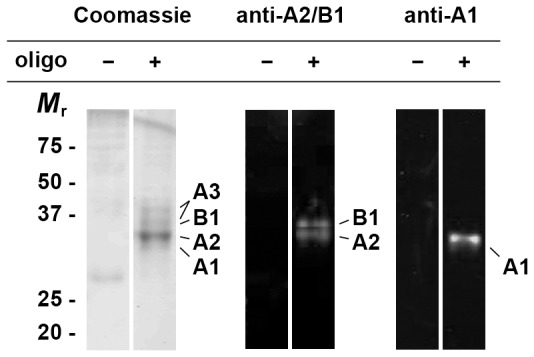
HnRNP A/B proteins in oligonucleotide pulldowns. Proteins pulled down from rat brain lysate using either unlabelled or dTelo4 oligonucleotide-labelled magnetic beads were resolved by SDS-PAGE and either stained with Coomassie Blue G250, or transferred to an Immobilon-Fl membrane for Western blotting. HnRNPs A1 and A2/B1 were identified by the application of anti-hnRNP A1 (Sigma, clone 4B10) followed by secondary anti-mouse IgG IRD800 or anti-rabbit A2/B1 [12] followed by secondary anti-rabbit IgG Alexa-680 fluorophore.

We first confirmed hnRNPs A1, A2 and A3 as the predominant proteins recovered from rat brain lysate through pulldowns with either the A2RE RNA trafficking recognition sequence, or using a single stranded DNA sequence containing four copies of the telomeric repeat (TTAGGG). Protein identities were confirmed by SDS/polyacrylamide gel electrophoresis and Western blotting (Fig. 1). A combination of mass spectrometric and Edman sequencing techniques were subsequently used to investigate post-translational modifications of the proteins isolated in these pulldowns.

Using comparative MALDI-TOF peptide mass fingerprinting of recombinant and native rat brain hnRNP A2 proteins, we initially identified a modification of +42 Da to N-terminal peptides of the native hnRNP A2 protein (see Supporting Information Fig. S1). The most likely explanation for this mass difference is acetylation of the N-terminal methionine and our earlier repeated but unsuccessful attempts to sequence hnRNP A2 by Edman degradation were consistent with the presence of an N-terminal modification. Acetylation of the N-terminal residues of both hnRNPs A2 and A3 was subsequently confirmed by LC-MS/MS (Table 1).

**Table 1:** Survey of post-translational modifications of the hnRNP A/B family proteins.

m/z ^[charge]^	M*_r_*	Sequence^a^	Peptide (enzyme^b^)	Arg	DMA	aDMA diagnostic ions^c^	Comments
*hnRNP A2*							
569.8 ^[2+]^	1137.6	MEREKEQF	1–8 (c’trypsin)				ESI, acetylation on Met-1
1313.6 ^[1+]^	1312.6	SGRGGNFGFGDSR	189–201 (trypsin)	191,201 188*			MALDI, cleavage after Arg-188 indicates absence of methylation
716.8 ^[2+]^	1431.6	DSRGGGGNFGPGPGSN	199–214 (t’lysin/AspN)	201			ESI
636.8 ^[2+]^	1271.6	FRGGSDGYGSGRG	215–227 (t’lysin/AspN)	216,226			ESI
651.3 ^[1+]^	650.3	GGGRGGY	251–257 (c’trypsin)		254	yes	MALDI
943.7 ^[3+]^	2828.1	FGGSPGYGGGRGGYGGGGPGYGNQGGGYGGGY	244–275 (t’lysin/AspN)		254		ESI, partial MMA and Arg detected in cultured cell lysates
*hnRNP A3*							
597.3 ^[3+]^	1788.9	MEVKPPPGRPQPDSGR	1–16 (trypsin)				ESI, acetylation on Met-1
526.9 ^[3+]^	1577.7	QSAGSQRGRGGGSGNF	209–223 (c’trypsin)		214,216		ESI
546.3 ^[4+]^	2181.2	SKQEMQSAGSQRGRGGGSGNF	203–223 (c’trypsin)		214,216	yes	ESI
391.7 ^[2+]^	781.4	FMGRGGN	223–229 (t’lysin)		226		ESI, Ox-met
591.2 ^[2+]^ 1181.5 ^[1+]^	1180.5	GGGGGNFGRGGNF	231–243 (c’trypsin)		239	yes	ESI MALDI
496.2 ^[3+]^	1485.6	GGRGGYGGGGGGSRGSY	244–260 (c’trypsin)	257	246		ESI
456.2 ^[2+]^ 911.4 ^[1+]^	910.4	GGGGGGSRGSY	250–260 (c’trypsin)	257			ESI MALDI
654.2 ^[1+]^	653.2	SSRGGY	284–289 (c’trypsin)		286	yes	MALDI
600.3 ^[2+]^ 1199.5 ^[1+]^	1198.5	SSRGGYGGGGPGY	284–296 (c’trypsin)		286		ESI MALDI
1832.6 ^[1+]^	1831.6	SSRGGYGGGGPGYGNQGGGY	284–303 (c’trypsin)		286	yes	MALDI
*hnRNP A1*							
551.5 ^[4+]^	2202.0	SKQEMASASSSQRGRSGSGNF	182–202 (c’trypsin)	196	194	yes	ESI
648.8 ^[2+]^	1295.6	GGGRGGGFGGNDNF	203–216 (c’trypsin)		206	yes	ESI
638.3 ^[3+]^	1911.9	GGGRGGGFGGNDNFGRGGNF	203–222 (c’trypsin)		206,218	yes	ESI
620.3 ^[2+]^	1238.5	GGNDNFGRGGNF	211–222 (c’trypsin)		218		ESI
610.3 ^[3+]^	1827.8	GGNDNFGRGGNFSGRGGF	211–228 (c’tryspin)		218,225		ESI
462.2 ^[2+]^	1383.7	SGRGGFGGSRGGGGY	223–237 (c’trypsin)		225,232	yes	ESI
660.0 ^[3+]^	1976.9	SGRGGFGGSRGGGGYGGSGDGY	223–244 (c’trypsin)		225,232	yes	ESI

a residues with a modified mass are underlined.

b t’lysin = thermolysin, c’trypsin = chymotrypsin.

c peak at m/z 46 or neutral loss of 45 Da.

### The hnRNP A/B Proteins Possess Distinct Methylation Profiles

Having accounted for N-terminal acetylation, mass discrepancies of ∼28 and ∼169 Da remained for hnRNPs A2 and A3, respectively. These values indicated that both proteins were further modified and given the previous evidence of hnRNP A/B dimethylation by protein arginine methyltransferases, suggested that hnRNPs A2 and A3 possess one and six dimethylarginine residues, respectively. For hnRNP A1, the other paralog belonging to this family, four sites of arginine dimethylarginine had previously been identified [14], [32].

*hnRNP A2*- We initially analysed endoproteinase digests of the recombinant and rat brain proteins to identify the remaining, covalently modified residue(s) in hnRNP A2. Tryptic peptides from recombinant and native rat brain hnRNP A2 protein were separated and analysed by electrospray ionization LC-MS (see Fig. S2A,B). Total ion current chromatograms revealed differences in peptide composition for the native and recombinant hnRNP A2 that ultimately were consistent with the protein being dimethylated (see Table S1). First, peak 15 in the *E. coli*-expressed protein included a peptide with a monoisotopic mass of 2494.4 Da, which was absent from the rat protein. This mass corresponded to a tryptic peptide covering residues 227–254. Likewise, peak 18 displayed a single average mass of 5081.2 Da for the *E. coli-*expressed protein, matching the theoretical value for a peptide comprising residues 255–305 (see Supporting Information Fig. S2C). No equivalent fragment was observed in the rat brain A2 digest. Finally, peak 19 (average mass 7590.0 Da), which was observed only in the rat brain A2 digest (see Supporting Information Fig. S2C), corresponded to a peptide of residues 227–305, suggesting that the rat protein, but not the recombinant protein, is modified in a way that eliminates tryptic cleavage at Arg-254. The mass of the peptide in peak 19 was 28 Da above the theoretical mass, consistent with dimethylation of this peptide, most likely at Arg-254.

For subsequent experiments, hnRNP A2 was digested firstly with thermolysin and then with endoproteinase AspN and subjected to analysis by LC-MS/MS. This strategy generated a peptide comprising residues 244–275, inclusive of Arg-254 and considered to be of a size more suitable for sequence analysis via fragmentation. The target peptide was successfully isolated and identified predominantly in triply charged form (m/z 943.7) (Table 1). Fragmentation data confirmed that this peak corresponded to the peptide covering residues 244–275 and showed conclusively that the Arg-254 residue contained a +28 Da modification.

Further experiments failed to identify any other sites of post-translational modification in hnRNP A2 (Table 1). This was not unexpected, as the previously measured mass discrepancy of ∼70 Da was completely accounted for by Met-1 acetylation (42 Da) and Arg-254 dimethylation (28 Da). Arg-254 is the final of six arginine residues within the putative RGG-box domain of hnRNP A2. RGG-box domains are characterized by the presence of a number of (Arg-Gly-Gly)-like repeats and the arginines in this context are common substrates for protein arginine methyltransferase activity. We confirmed that the other five arginine residues of the hnRNP A2 RGG-box domain (i.e. Arg-188, Arg-191, Arg-201, Arg-216 and Arg-226) were not dimethylated, again using MALDI-TOF peptide mass fingerprinting (see Fig. S3). Enzymatic digestion with endoproteinase Asp-N produced three peptides that collectively included the five target arginine residues and all three peptides were detected at their expected m/z in the mass spectrum: no peptide masses indicative of one (or multiple) +28 Da modifications were detected.

*hnRNP A3*- To parallel the experiments performed for hnRNP A2, LC-MS/MS analysis was also used to identify post-translationally modified residues within hnRNP A3. In the case of A3, LC-MS/MS analysis followed prior enzymatic digestion with thermolysin, trypsin or chymotrypsin. Peptides that yielded information supporting post-translational modification of hnRNP A3 are listed in Table 1, with six arginine residues identified as potential sites of post-translational dimethylation. Subsequent fragmentation analysis confirmed that Arg-214, Arg-216, Arg-226, Arg-239, Arg-246 and Arg-286 are all dimethylated. The presence of six dimethylated arginine residues (in addition to the previously identified N-terminal acetylation) was again consistent with the earlier observed mass discrepancy of ∼211 Da.

*hnRNP A1*- We subsequently employed this same strategy to re-examine the methylation profile for hnRNP A1. In addition to confirming dimethylation of Arg-194, Arg-206, Arg-218 and Arg-225, we identified a previously unreported dimethylarginine at residue 232 (Table 1). This brought the total number of dimethylated arginine residues within the RGG box region of hnRNP A1, to five, leaving only one arginine site (Arg-196) unmethylated.

At this point, it was apparent that the pattern of hnRNP A2 methylation contrasted remarkably with that of hnRNP A1, as well as the now established pattern of methylation for hnRNP A3. The importance of the finding that hnRNP A2 is modified on a single residue, however, is underscored in that it now provides a convenient model system to study the biological consequences of arginine methylation (e.g. through the generation of a single, site-directed mutant). For this reason, our immediate further studies were focused on hnRNP A2.

### HnRNP A2 is Asymmetrically Dimethylated

**Figure 2:**
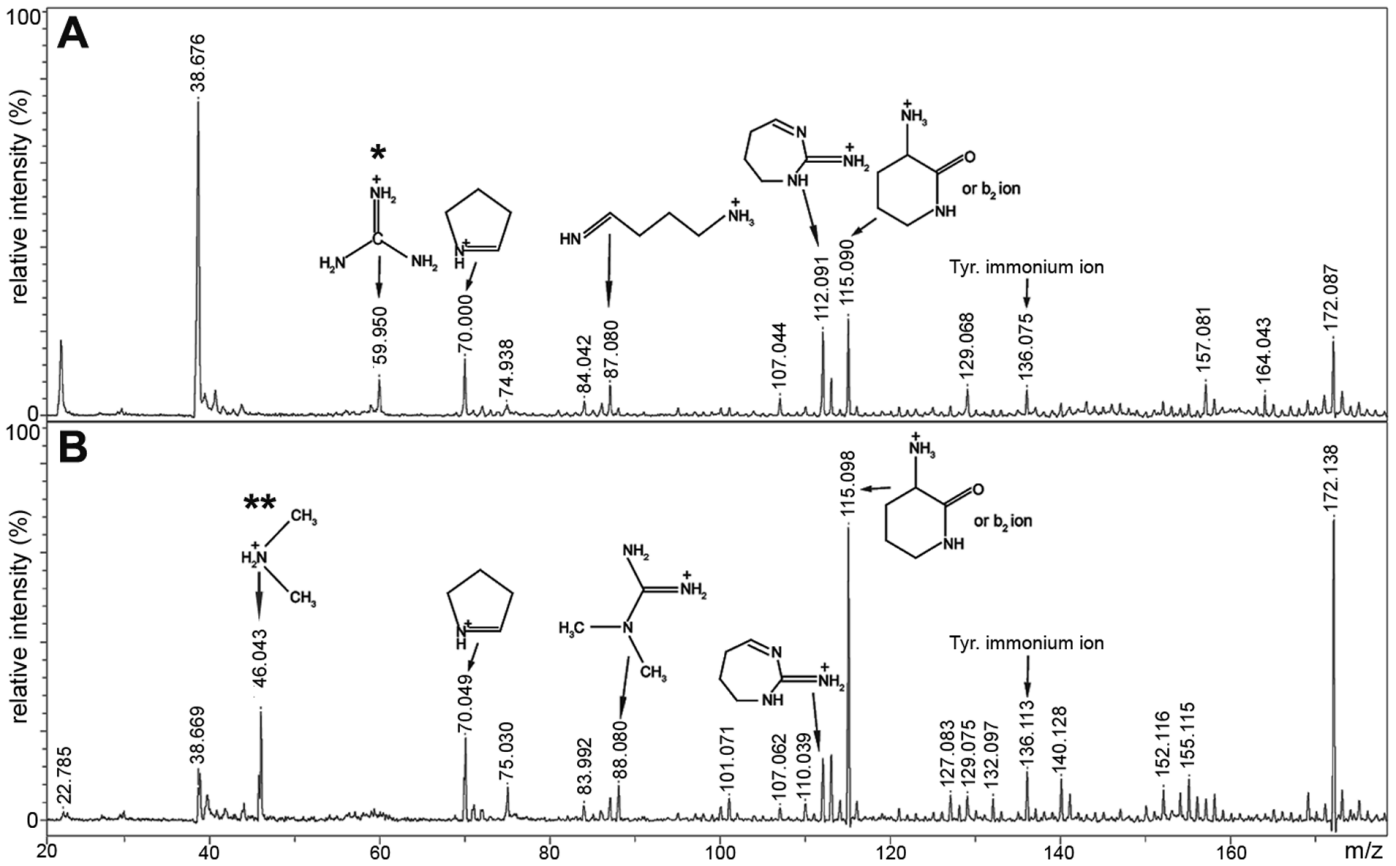
Detection of diagnostic arginine and asymmetric dimethylarginine ions. Zoomed view from region m/z 20–175 of MALDI-TOF/TOF analysis of (A) m/z 623.2, corresponding to unmodified peptide 251-GGGRGGY-257 from recombinant hnRNP A2 and (B) m/z 651.3, corresponding to peptide 251-GGGRGGY-257 with a dimethylarginine modification from HPLC-purified rat brain hnRNP A2. Structures representing diagnostic ions of arginine* and dimethylarginine** are labeled as previously described [30]. See Supporting Information Fig. S4 for full spectrum.

Two distinct families of protein arginine methyltransferases exist, distinguished by the way in which the arginine sidechains are modified. Type I PRMTs asymmetrically place both methyl groups on a single guanidino nitrogen atom to produce *N* ^G^,*N* ^G^-dimethylarginine while type II PRMTs symmetrically place a single methyl group on each guanidino nitrogen atom resulting in *N* ^G^,*N* ^G^’-dimethylarginine. We were able to distinguish between asymmetric dimethylarginine (aDMA) and symmetric dimethylarginine (sDMA) by interrogating MALDI-TOF/TOF fragmentation spectra acquired following collision-induced dissociation. Peptides generated by chymotrypsin digestion of HPLC-purified recombinant or rat brain A2 were identified at m/z 623.2 (for recombinant A2) or m/z 651.28 (for rat brain A2) by MALDI-TOF mass spectrometry. These ions corresponded to the peptide 251-GGGRGGY-257, either unmodified or +28 modified, respectively. When fragmented by MS/MS, both peptides yielded diagnostic ions that revealed the modification status relating to Arg-254 (Fig. 2 and Fig. S4). Fragmentation of the peptide from recombinant A2 (m/z 623.2) produced diagnostic ions consistent with arginine (Fig. 2A), while the modified peptide from rat brain A2 (m/z 651.28) generated diagnostic ions for aDMA, including the dimethylammonium ion at m/z 46.06 (Fig. 2B), with neutral losses of dimethylamine (45 Da) from the precursor and fragment ions also observed.

**Figure 3:**
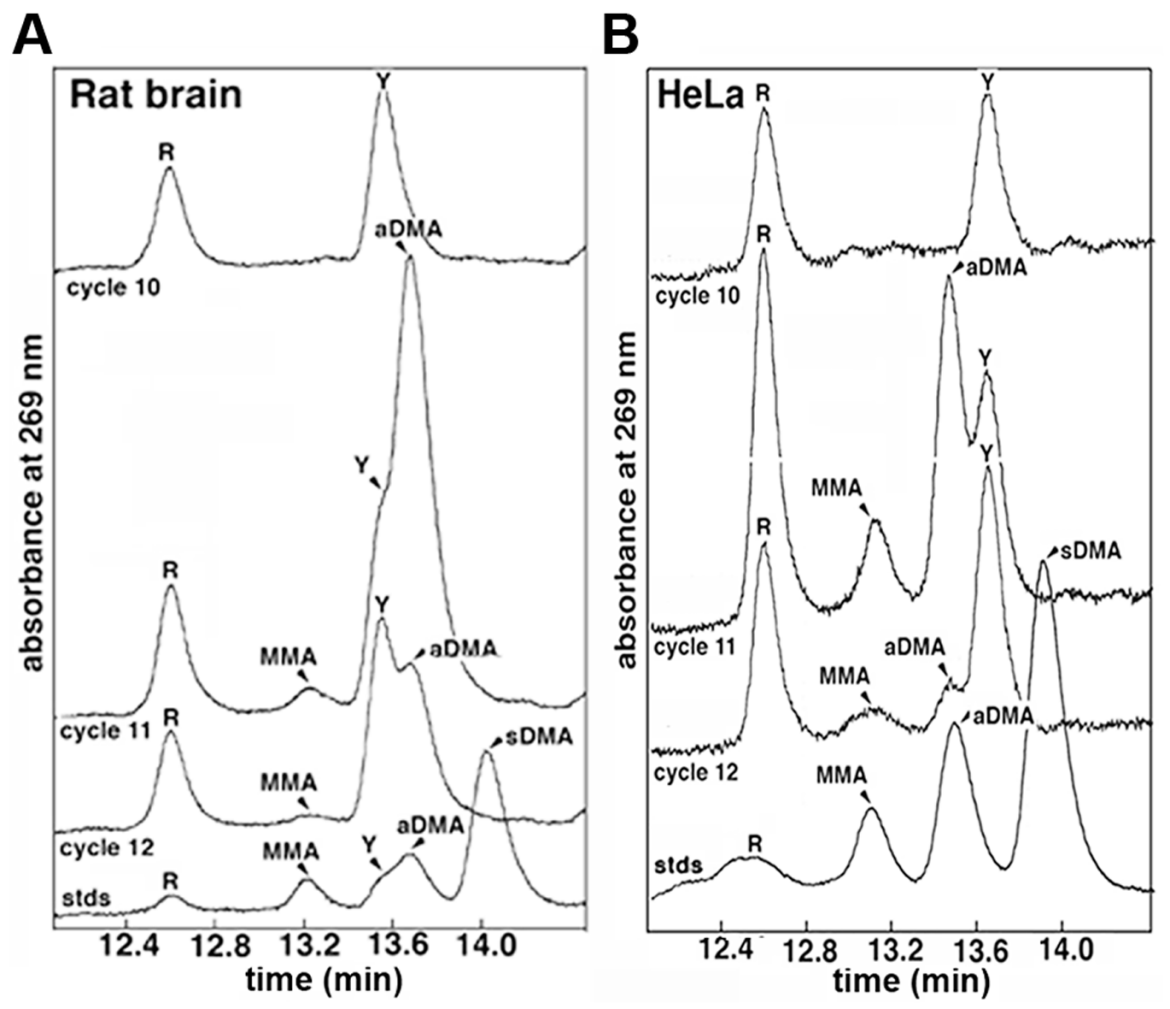
Edman degradation detects methylated arginine residues. Elution profiles from rat brain (A) and HeLa cell hnRNP A2 (B) showing degradation cycles 10–12 aligned with standards (stds) containing a mixture of unmodified (R), monomethylated (MMA), asymmetrically dimethylated (aDMA), and symmetrically dimethylated (sDMA) arginine residues. In each case, the residue released in cycle 11 is residue 254. The retention time of the residue released in this cycle indicates that residue 254 of hnRNP A2 is aDMA (i.e. *N* ^G^,*N* ^G^–dimethylarginine). In both cases, a proportion of unmodified arginine and MMA is observed that is small in the rat brain protein but significant for the protein isolate from HeLa cells. No sDMA is detected in either sample. The retention time of amino acids was determined by comparison to chemically pure standards (trace labelled “stds” at the bottom of each panel). The transposition of retention times observed for aDMA and tyrosine (Y) between panels A and B was caused by a small change in the HPLC buffer.

Asymmetric dimethylation of hnRNP A2 was independently confirmed via Edman degradation analysis of a proteolytic fragment of rat brain hnRNP A2 comprising residues 244–275. Edman data obtained for cycles 1–10 matched the anticipated sequence for the first 10 residues of the peptide (i.e. residues 244–253; Fig. 3A). The residue in cycle 11 (the expected position of Arg-254) was unambiguously aDMA. No sDMA was detected in any of the cycles, whereas in cycle 11 the aDMA increased markedly above the cycle 10 level, and dropped in cycle 12.

### HnRNP A2 is Differentially Methylated Across Cell Types

Unexpectedly, our Edman degradation analysis suggested a difference in the extent of hnRNP A2 methylation between protein recovered from rat brain tissue homogenate (Fig. 3A) and protein isolated from cultured human epithelial (HeLa) cells (Fig. 3B). Edman analysis of rat brain protein revealed a small arginine signal in the cycle coincident with residue 254. The fact that this signal remained relatively constant throughout cycles 10, 11 and 12 suggested that little of residue 254 was unmodified arginine and that the bulk of the signal from this residue could be attributed to asymmetric dimethylarginine, as outlined above. When hnRNP A2 isolated from HeLa cells was analysed in the same way, however, the signal in cycle 11 indicated appreciably higher levels of both unmodified and monomethylarginine (MMA), in addition to the predominant asymmetric dimethylarginine (Fig. 3B). This observation suggested that arginine and MMA may be more abundant in A2 extracted from HeLa cells than from rat brain.

**Figure 4:**
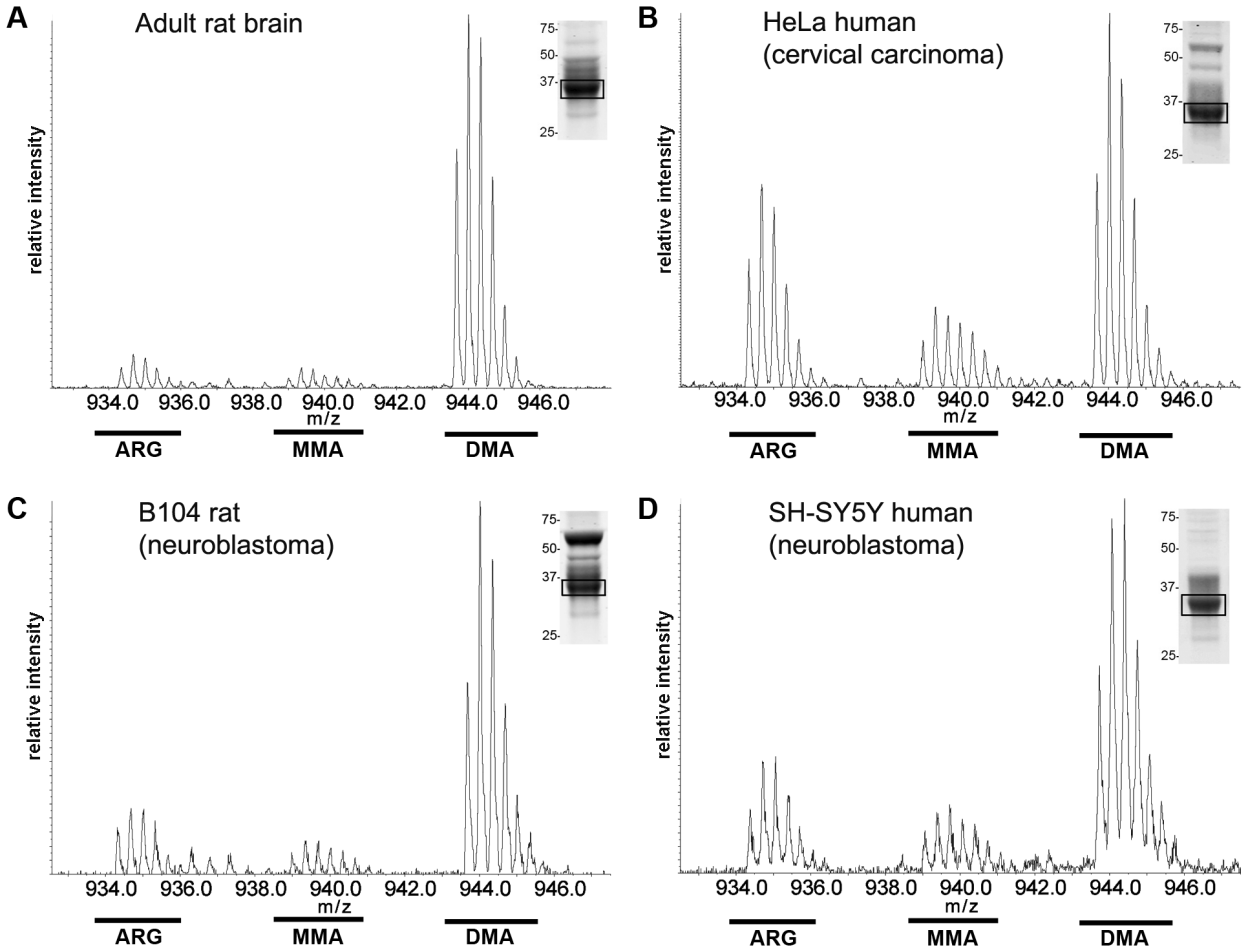
LC-MS/MS identification of monomethylated and unmodified Arg-254 residues in hnRNP A2 sourced from different cell types. Comparative LC-MS/MS analysis of the peptide 244-FGGSPGYGGGRGGYGGGGPGYGNQGGGYGGGY-275, derived from hnRNP A2 protein isolated by pulldown assay (boxed band indicated in representative Coomassie-stained gel lanes, shown as insets). The modified peptides were obtained by thermolysin/AspN double digestion of endogenous protein purified from either (A) rat brain tissue homogenate, (B) HeLa, (C) B104 or (D) SH-SY5Y cultured cell lysates. The position of ion series indicative of either unmodified Arg, MMA or DMA at position Arg-254 is shown. The box identifies the band extracted for digestion and LC-MS/MS analysis. A cluster of peaks (rather than a single ion) is observed at each m/z, due to the natural abundance of heavy isotopes (predominantly ^13^C).

This finding was confirmed when LC-MS/MS spectra, recorded during purification of the peptide subjected to Edman degradation, were analysed. Spectra recorded for rat brain hnRNP A2 showed a predominant isotopic m/z envelope series centred at around 943.7, the expected m/z for the triply charged, dimethylated peptide (Fig. 4A). Only very weak isotope series were recorded at the expected m/z for unmethylated and monomethylated peptide, respectively. Comparatively, the peptide generated from HeLa-sourced hnRNP A2 showed substantially greater signal at the expected m/z for the triply charged, unmethylated peptide and to a lesser extent for the monomethylated peptide (Fig. 4B). We estimated from the combined Edman degradation and LC-MS/MS analyses that as much as 50% of the HeLa cell hnRNP A2 population could be accounted for by either unmethylated or monomethylated protein.

We extended this analysis to investigate whether the variation in relative amounts of unmodified arginine to MMA and aDMA at residue 254 seen in rat brain and HeLa cell A2 was mirrored in other cell types. In the same way, hnRNP A2 Arg-254-containing peptides were examined for proteins isolated from total cell lysates of both rat (B104) and human (SH-SY5Y) neuroblastoma cell lines, for comparison to the rat brain and HeLa cell proteins (Fig. 4C,D). We found that in both cases, hnRNP A2 obtained from immortalized cells had higher concentrations of unmodified and monomethylated Arg-254 than those observed in rat brain (compare Fig. 4A with Fig. 4C,D), although neither protein appeared to be unmethylated to the same extent as protein sourced from HeLa cells (Fig. 4B). Taken together, these data suggest that hnRNP A2 arginine methylation may be differentially regulated between immortalized cell lines and primary cells sourced from rat brain.

### Methylation of hnRNP A2 does not Influence its Nucleocytoplasmic Distribution

Previous studies suggested that arginine methylation within the RGG box of hnRNP A2 may affect its nucleocytoplasmic distribution [18], [19], [33]. To investigate this hypothesis, an expression construct was generated that yielded GFP-tagged hnRNP A2 containing an Arg to Ala point mutation at position 254 (A2^R254A^). As Arg-254 is the sole methylated residue within hnRNP A2, this construct allowed us to compare side by side the localization of methylated and non-methylated forms of hnRNP A2, without the need to employ chemical inhibitors used in previous studies that may widely and/or unpredictably influence the cellular steady state. For these studies, we employed the three cell lines for which methylation of endogenous hnRNP A2 had been established (HeLa, B104 and SH-SY5Y). We reasoned that an altered subcellular distribution could be expected for the A2^R254A^-GFP, compared to A2-GFP, if the hypothesis that dimethylation regulates nucleocytoplasmic distribution was correct.

**Figure 5:**
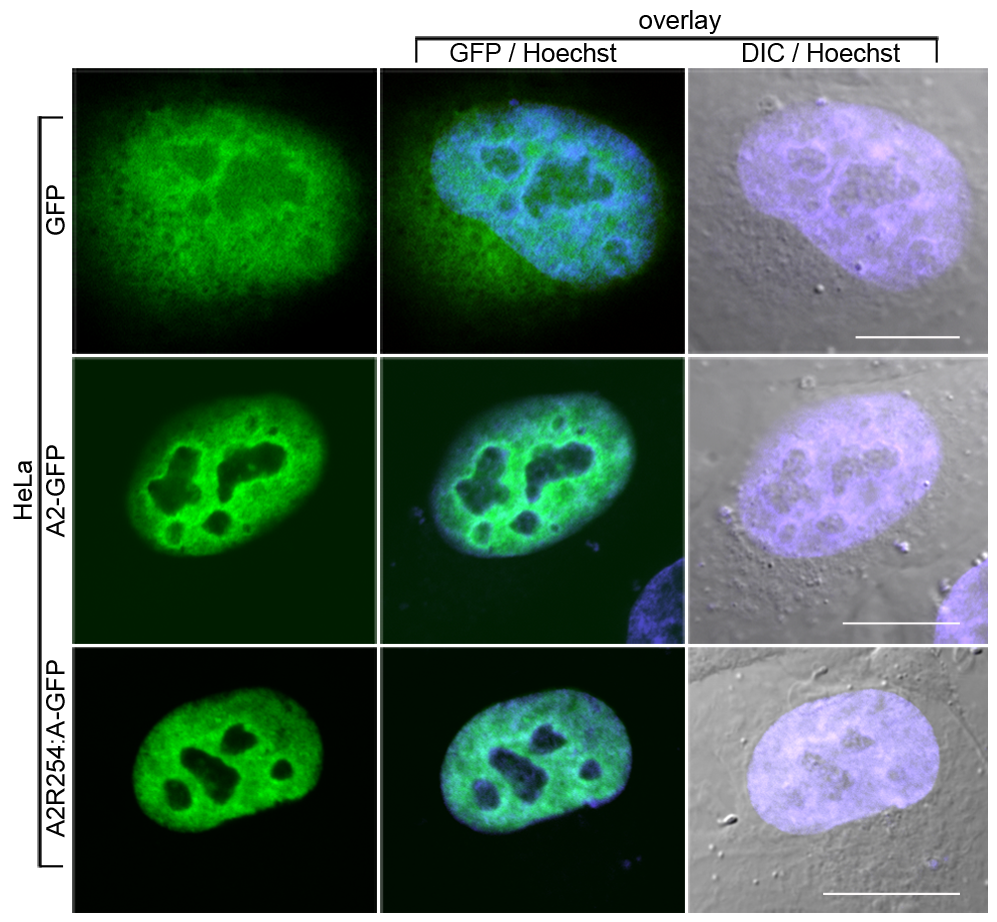
Nuclear localization of transiently expressed wild type and A2^R254A^ in interphase HeLa cells. HeLa cells were transfected with GFP vector (first row) wild-type A2-GFP (second row) or A2^R254A^-GFP. For the control vector, GFP is diffusely localized throughout out the nucleus and extranuclear region. Both A2-GFP and the A2^R254A^-GFP point mutant, which lacks the sole methylated arginine residue, are exclusively observed in the nucleus. The signal from Hoechst dye (blue) is overlaid on both the GFP signal (centre panels) and images obtained by digital interference contrast (DIC) microscopy (right panels) to highlight the location of the nucleus. Scale bar = 10 µm.

In accord with previous work [10], control HeLa cells transiently transfected with pEGFP showed a diffuse pattern of GFP expression throughout the cells (Fig. 5). In comparison, cells transiently transfected with the wild type A2-GFP construct exhibited a signal that was overwhelmingly nuclear (Fig. 5). Surprisingly, HeLa cells expressing A2^R254A^-GFP also showed a nuclear signal indicating that the unmethylated point mutant was confined to the nucleus (Fig. 5). This phenotype was invariant across all interphase cells imaged and identical results were obtained when these experiments were repeated in both B104 and SH-SY5Y cells (see Fig. S5), demonstrating that the absence of dimethylarginine, for all three cell lines tested here at least, neither directs hnRNP A2 to the cytoplasm nor influences its nuclear retention. Preliminary observations employing primary oligodendrocytes and hippocampal neurons cultured from rats are also consistent with this phenotype (data not shown).

**Figure 6:**
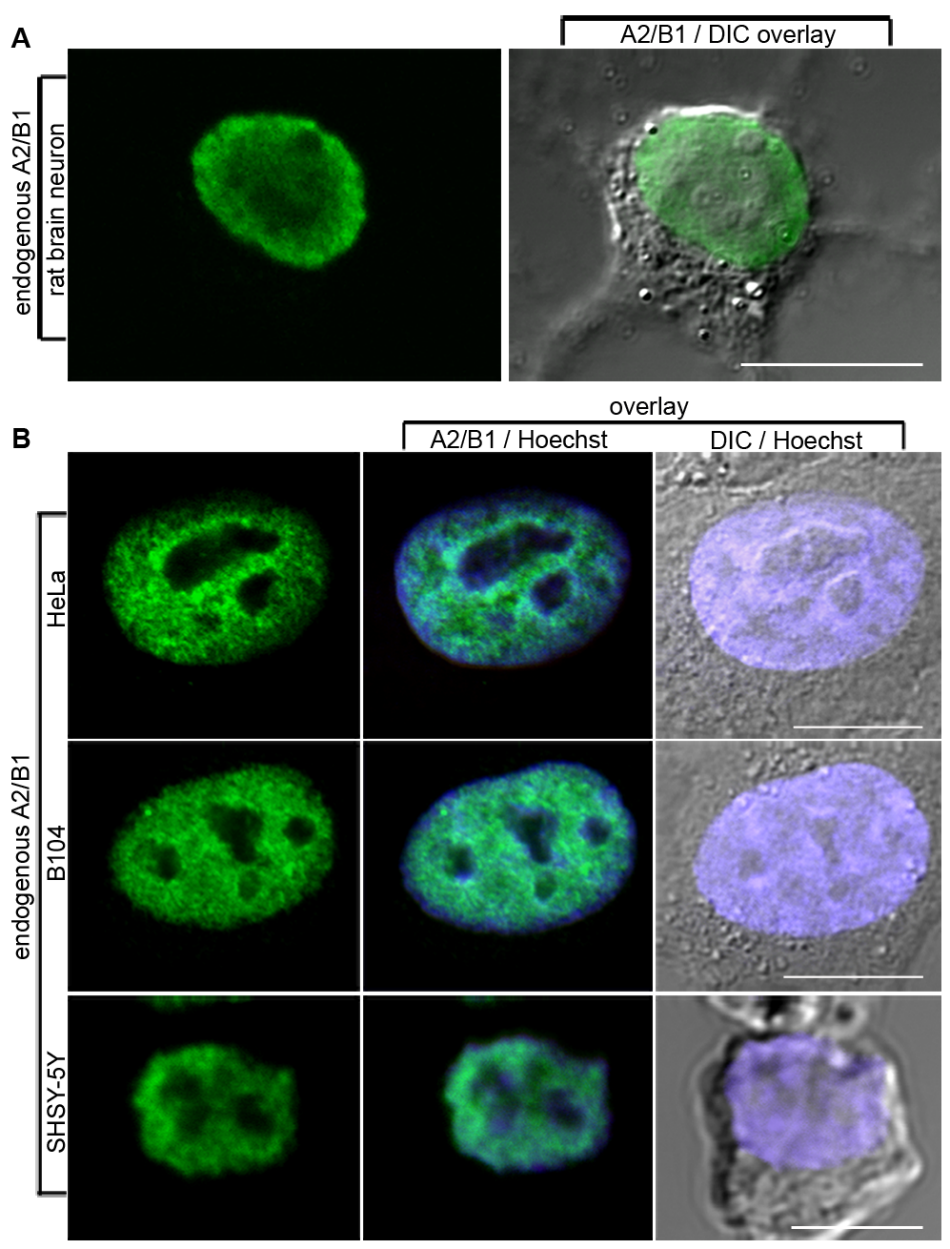
Nuclear localization of endogenous methylated and unmethylated hnRNP A2 in interphase cells. Endogenous A2/B1 in primary (A) rat brain hippocampal neurons and (B) HeLa, B104 and SH-SY5Y cells was stained with rabbit polyclonal A2/B1 antibody followed by anti-rabbit Alexafluor-488-conjugated secondary antibody and imaged by confocal microscopy. While the four cell systems imaged contain variable proportions of unmethylated, monomethylated and dimethylated hnRNP A2, all cells show an exclusively nuclear pattern of localization with no signal corresponding to hnRNP A2 detected outside the nucleus. Nuclear staining (Hoechst dye) and DIC images (B only) are overlaid. Scale bar = 10 µm for HeLa and B104, 5 µm for SH-SY5Y.

To support these findings, we studied the endogenous protein distribution of hnRNP A2 via immunofluorescence and made use of our finding that in the cell lines examined here, significant pools of unmethylated and monomethylated hnRNP A2 exist (Figs. 3–4). Again, HeLa, B104 and SH-SY5Y cells were employed, and immunostaining was carried out using an antibody that detects A2 independently of the methylation status [12]. By this method, endogenous hnRNP A2 was found to be overwhelmingly localized to the nuclei not only of B104 and SH-SY5Y cells (Fig. 6B) but also in HeLa cells (Fig. 6B) where, as ascertained previously, a significant pool of unmethylated A2 exists. Again the phenotype was invariant across all cells imaged (see Fig. S6). Primary rat brain neurons, where Arg-254 of hnRNP A2 is almost completely dimethylated (Fig. 3A), were analysed in the same way (Fig. 6A) with the finding again that, at the level of detection by immunofluorescence, hnRNP A2 is exclusively localized to the nucleus.

## Discussion

**Figure 7:**
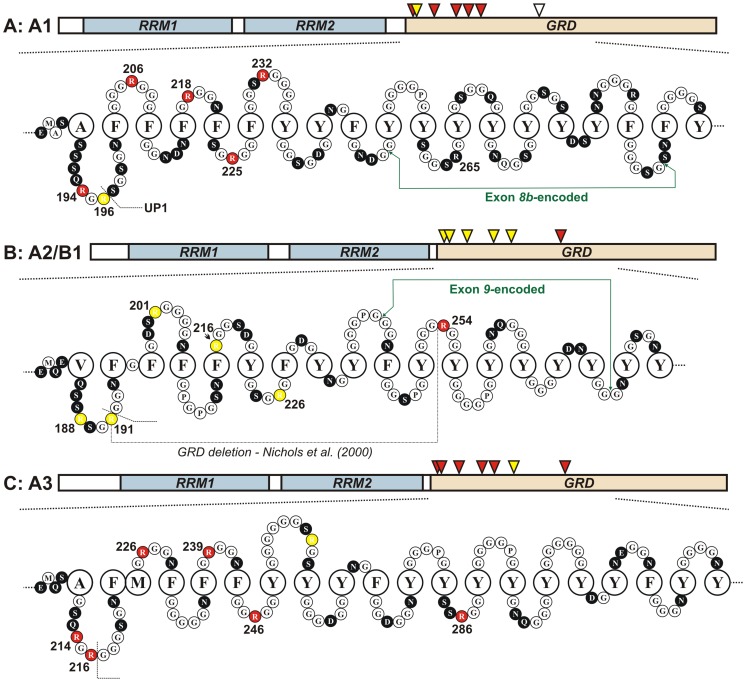
A comparison of arginine methylation between the A/B hnRNPs. Represented schematically are magnified views of the RGG box domains of hnRNPs A1, A2 and A3, respectively (A–C). Each representation begins with the residues that lie immediately outside the second RRM and includes residues encoded by the alternatively spliced exons *8b* and *9* for hnRNPs A1 and A2/B1 and the corresponding residues of hnRNP A3. The sequences are shown as “omega loops”, expanding on a structure first proposed by Steinert *et al*. [39]. While these structures are hypothetical, biophysical data consistent with the GRD adopting such a fold have been reported for both hnRNP A2 [26] and other RNA-binding, Gly-rich proteins [40]. Large open circles represent hydrophobic residues; small black circles represent hydrophilic residues; small, hydrophobic residues (predominantly glycine) are represented by small open circles. Sites of modified arginine residues (red) for A1, A2 and A3 have been annotated from the findings discussed in this study: many of these sites are on or close to the apex of the glycine loop motif. Unmethylated arginine residues are coloured yellow. The position labelled “UP1” indicates the cleavage site that generates unwinding protein 1 from hnRNP A1. The corresponding residue is similarly demarcated for hnRNP A2 while for hnRNP A3, Arg-216 is aDMA-modified and thus unlikely to be cleaved by the trypsin-like protease. Residue numbering reflects the most abundant isoform hnRNP A2 (NCBI: NP_112533) and the full length isoforms hnRNP A1^B^ (NCBI: P09651) and hnRNP A3^A^ (NCBI: P51991).

Post-translational *N* ^G^,*N* ^G^-arginine dimethylation of nuclear hnRNPs is evolutionarily conserved from lower eukaryotes to mammals [17]. The majority of this methylation is attributable to proteins belonging to the hnRNP A/B subfamily and although first detected over three decades ago [2], [15], [24], the functional consequences of arginine dimethylation remain unclear. Prior to this study, it had been postulated that post-translational arginine methylation regulates the nucleocytoplasmic distribution of hnRNP A2 [19] since deletion of most of the RGG box region (Fig. 7B), results in a four-fold increase in the cytoplasmic-to-nuclear ratio of the shortened protein [19] while treatment with adenosine dialdehyde (AdOx), an indirect inhibitor of arginine methyltransferase activity, reportedly results in an altered ratio consistent with redistribution of hnRNP A2 from the nucleus to the cytoplasm [19].

Our findings are contrary to this hypothesis. The simplicity of the hnRNP A2 system, where only a single arginine is modified, allowed us to investigate the influence of arginine methylation on subcellular localization using a transiently-expressed point mutant conjugated to GFP (A2^R254A^). These experiments revealed an exclusively nuclear pattern of A2^R254A^ localization, identical to wild type hnRNP A2, but distinct from control cells transfected with GFP alone. We independently validated this observation by analysing the pattern of localization of hnRNP A2 via immunostaining of the endogenous protein population in primary cultured neurons, as well as in three different immortalized cell lines (human HeLa, SH-SY5Y and rat B104). Amongst these four systems, the extent of arginine methylation at residue 254 was found to vary from 50 to almost 100%, but despite the existence of up to 50% unmethylated hnRNP A2 (in HeLa cells) the localization of hnRNP A2 in all three cell lines tested, as well as in primary rat brain neurons, was found to be exclusively nuclear. Our findings are also consistent with data obtained by fluorescence correlation spectroscopy analysis of primary oligodendrocytes that had been microinjected with constructs expressing A2-GFP or A2^R254A^-GFP; no significant difference was observed between the cytoplasmic-to-nuclear (C:N) ratio of the two GFP fusion constructs (Friend et al. *unpublished observations*). Furthermore, an earlier analysis of the subcellular localization of hnRNP A2/B1 in cycling HeLa cells also showed that these proteins are characteristically confined to the nucleus throughout interphase [25]. It is therefore apparent that the presence or absence of methyl groups at residue 254 alone is not sufficient to regulate the nucleocytoplasmic distribution of hnRNP A2, although we cannot at this stage rule out the possibility that the RGG box regulates nucleocytoplasmic shuttling through some other mechanism, directly or otherwise.

Importantly, the authors of the earlier study correlating arginine methylation with reduced nuclear retention of hnRNP A2 [19] noted that the outcome of their experiments was heavily dependent on the concentration of AdOx used as well as cell cycle stage. However a contrary result was observed in yeast, where an earlier report had suggested that in the *absence* of methylation, nuclear export of hnRNP A/B homologs is impaired [20]. Entirely consistent with our observations, however, are the more recent findings of an independent study [34], which failed to find any effect on the subcellular distribution of hnRNP A2 following treatment of primary rat oligodendrocytes with either AdOx or with an alternative, competitive inhibitor of protein methyltransferases, 5,6-dichloro-1-β-D-ribofuranosylbenzimidazole. There are also parallels with hnRNP K which contains an RGG box and 5 asymmetrically dimethylated arginine residues. Here methylation had no effect on subcellular localization and instead appeared to be dependent on either of two distinct bi-directional signals, one of which resembles the M9 sequence of hnRNPs A1 and A2 35,36.

In the current study, we have examined and compared the prevalence of dimethylarginine amongst members of the hnRNP A/B family (Fig. 7). Consistent with earlier findings [14], [31], we found the extent of arginine methylation in hnRNP A1 is relatively high, with a total of five out of the six RGG-like repeats that comprise the RGG box of hnRNP A1 being asymmetrically dimethylated on the arginine residue. Similarly, hnRNP A3 is highly methylated with six out of a possible seven RGG-like repeats asymmetrically dimethylated. However, a striking contrast was seen with hnRNP A2 and the alternative spliceform B1 (see Table S2), which we found to be methylated on only a single arginine residue (Arg254). Our findings are consistent with the earliest studies reporting methylation of hnRNPs A1 and A2 [31] and raise questions as to the biological relevance of a cellular repertoire of differentially methylated hnRNP A/B paralogs. The capacity to express multiple hnRNP A/B paralogs (and further, multiple alternatively spliced isoforms of each paralog), each with a different pattern of arginine methylation, suggests that methylation may in some way contribute to the observed functional differences amongst these proteins.

While the functional role of arginine methylation within the hnRNP A/B family remains uncertain, the proposition that methylation of these proteins may be involved in processes not directly related to localization is not without support [13], [14]. Earlier hypotheses have focused on a role for methylation in regulating the binding of nucleic acids, given that the consensus motifs targeted by arginine methyltransferases are exclusively localized within the RGG box domain, a domain generally associated with nucleic acid binding [13], [14], [16]. Consistent with this model, *in vitro* enzymatic methylation of arginine in recombinant hnRNP A1 has been shown to reduce its binding to single-stranded nucleic acids [16]. Outside of the hnRNP A/B family, recent studies have demonstrated that NAB1 binding to target mRNAs is regulated by methylation of its RGG box domain [37].

Indeed, evidence suggests that methylation of arginine side chains can modulate affinity for cognate nucleic acids and in turn regulate biological activity [17]. Given the capacity for arginine side chains to participate in H-bonding and the significant role played by H-bonding interactions in nucleic acid recognition, a role for RGG box methylation in regulating RNA binding would not be surprising. The addition of a methyl group to the guanidino nitrogen of arginine has no effect on the net charge of the side chain, but does reduce its potential for hydrogen bonding, suggesting that methylation may act to modulate nucleic acid binding, for example, through a reduced ability to participate in hydrogen bonding [17], [22], [23]. We therefore propose that the differing patterns of arginine methylation observed between hnRNPs A1, A2 and A3 (Fig. 7) may represent a means by which the RNA-binding activity [25] of these paralogs is regulated.

In summary, the hnRNP A/B paralogs have high sequence identity and are commonly considered to have parallel biological functions. However, we have shown that the pattern of post-translational modification of hnRNP A2 varies markedly compared to hnRNPs A1 and A3. Molecular mechanisms connecting modification of arginine residues to function are beginning to emerge [38] and the conservation and regulation of methylation of Arg-254 in hnRNPA2, as found in the current study, may impart functional diversity upon the hnRNP A/B proteins. The findings presented in this work strongly counter the existing hypothesis that hnRNP A/B protein arginine methylation governs their nucleocytoplasmic distribution and while the precise functional significance of dimethylation in the context of the hnRNP A/B proteins remains to be determined, it is the focus of ongoing work.

## Supporting Information

Figure S1 **Post-translational N-terminal acetylation of rat brain hnRNP A2 identified by MALDI-TOF mass spectrometry.** Recombinant (A, C) and rat brain hnRNP A2 (B, D) were digested with trypsin (A, B) or endoproteinase LysC (C, D). The unmodified peptides containing residues 1–3 (m/z 435.28 in A) and 1–5 (m/z 692.43 in C) were detected in the recombinant protein only whereas the corresponding peptides with +42 Da mass were observed only in the rat brain protein (m/z 477.26 in B and m/z 734.49 in D). Relative intensity is plotted as counts/s on the y-axis.
(TIF)

Figure S2 **HnRNP A2 peptide mass spectra reveal dimethylation of Arg-254.** (A & B) Proteins purified and digested with trypsin were separated and analyzed by LC-MS. Total ion current is plotted against the HPLC retention time. (C) Spectra of selected peaks that reveal a difference between recombinant and rat brain proteins. Only the latter generates a peptide of average mass 7590.0 Da (left panel, C), corresponding to residues 227–305 (peak 19 in A): the recombinant protein spectrum lacks this peak but has an additional peak containing a peptide with average mass 5081.2 Da (right panel in C) arising from residues 255–305 (peak 18 in B) as well as an additional mass of 2495.1 Da in peak 15 (corresponding to residues 227–254).
(TIF)

Figure S3 **MALDI-TOF mass spectra of AspN peptides from rat brain hnRNP A2 confirm other RGG-like motifs are unmodified.** The peaks arise from the peptides (A) 183–198 (containing Arg-188 & Arg-191), (B) 199–219 (containing Arg-201 & Arg-216) and (C) 220–229 (containing Arg-226). The asterisks mark the positions expected for dimethylation at each arginine residue (+28 or +56 Da). The minor peaks close to the right-hand asterisks in the left and center panels are outside the bounds of accuracy for +28 Da.
(TIF)

Figure S4 **Diagnostic ions reveal asymmetric dimethylation of Arg-254.** MALDI-TOF/TOF analysis of (A) m/z 623.2, corresponding to unmodified peptide 251-GGGRGGY-257 from chymotrypsin digest of recombinant A2 and (B) m/z 651.3 corresponding to peptide 251-GGGRGGY-257 with dimethylarginine modification from HPLC-purified rat brain hnRNP A2. Identified ions are indicated (refer to Fig. 2 of main text for additional details). DMG = dimethylated guanidinium, DMA = dimethylamine.
(TIF)

Figure S5 **Transfected A2 point mutant in SH-SY5Y, B104.** Transiently expressed A2^R254A^-GFP is exclusively localized to the nucleus of both B104 (upper panels) and SH-SY5Y transfected cell lines. Transfected GFP vector alone results in a diffuse nuclear and cytoplasmic signal. The nucleus is stained with Hoechst dye (blue) and is overlaid on the GFP signal (middle panels) and on corresponding DIC micrographs (right panels). Scale bar is 10 µm for B104 cells and 5 µm for SH-SY5Y cells.
(TIF)

Figure S6 **Field of view of endogenous A2 localized in the nucleus of HeLa, B104 and SH-SY5Y cells.** Cells were fixed and immunostained using a rabbit polyclonal antibody against hnRNP A2/B1, followed by an anti-rabbit Alexafluor-488-congugated secondary antibody. The DNA was stained using Hoechst dye (blue) and the cells were imaged using confocal microscopy and DIC. The far right panel represents the profile of subcellular staining of overlaid A2/B1 / Hoechst examined using Zeiss LSM meta510 software. A dotted line for each cell type (A2/B1/Hoechst overlay panels) indicates the profile examined. Scale bar is 10 µm for HeLa and B104 cells and 5 µm for SH-SY5Y cells.
(TIF)

Table S1 **Selected mass spectrometry data for tryptic peptides of hnRNP A2 overexpressed in** ***E. coli*** **(recombinant) and isolated from rat brain.** Mass spectrometry data for peaks from the LC chromatogram shown in Fig. S2A & S2B. Each peak confirms either the presence or absence of dimethylarginine for one of the five arginines highlighted for hnRNP B1 in yellow or magenta, respectively, in Fig. 7.
(DOC)

Table S2 **Post-translational modification of hnRNP B1.**
(DOC)
