# Supplementary figures and images for: Arginine Methylation of hnRNP A2 Does Not Directly Govern Its Subcellular Localization
Source: PLoS One. 2013 Sep 30;8(9):e75669. doi: 10.1371/journal.pone.0075669 (PMC3787039; doi:10.1371/journal.pone.0075669)

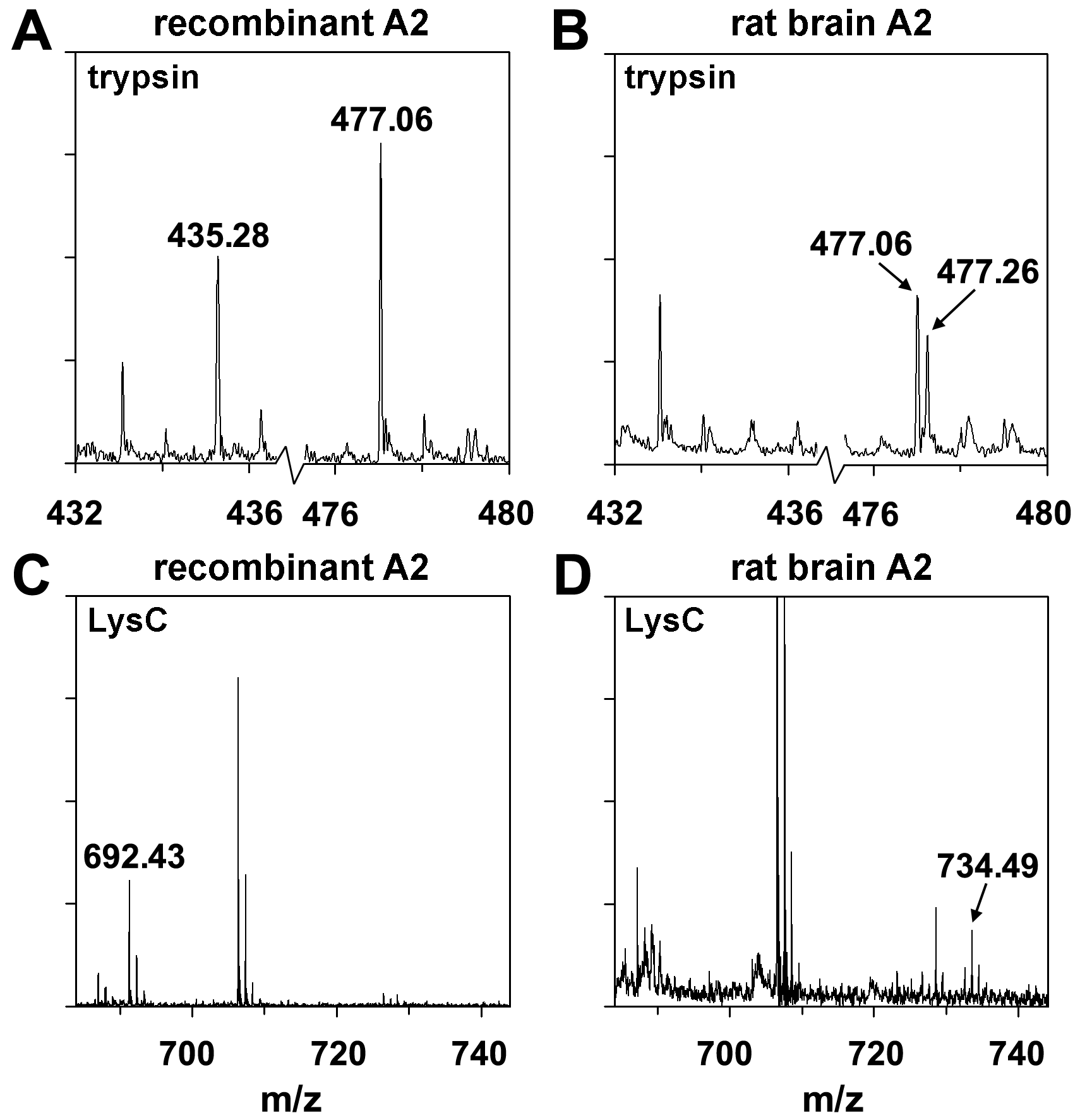

Supplement: Figure S1 — Post-translational N-terminal acetylation of rat brain hnRNP A2 identified by MALDI-TOF mass spectrometry. Recombinant (A, C) and rat brain hnRNP A2 (B, D) were digested with trypsin (A, B) or endoproteinase LysC (C, D). The unmodified peptides containing residues 1–3 (m/z 435.28 in A) and 1–5 (m/z 692.43 in C) were detected in the recombinant protein only whereas the corresponding peptides with +42 Da mass were observed only in the rat brain protein (m/z 477.26 in B and m/z 734.49 in D). Relative intensity is plotted as counts/s on the y-axis. (TIF) [file pone.0075669.s001.tif]

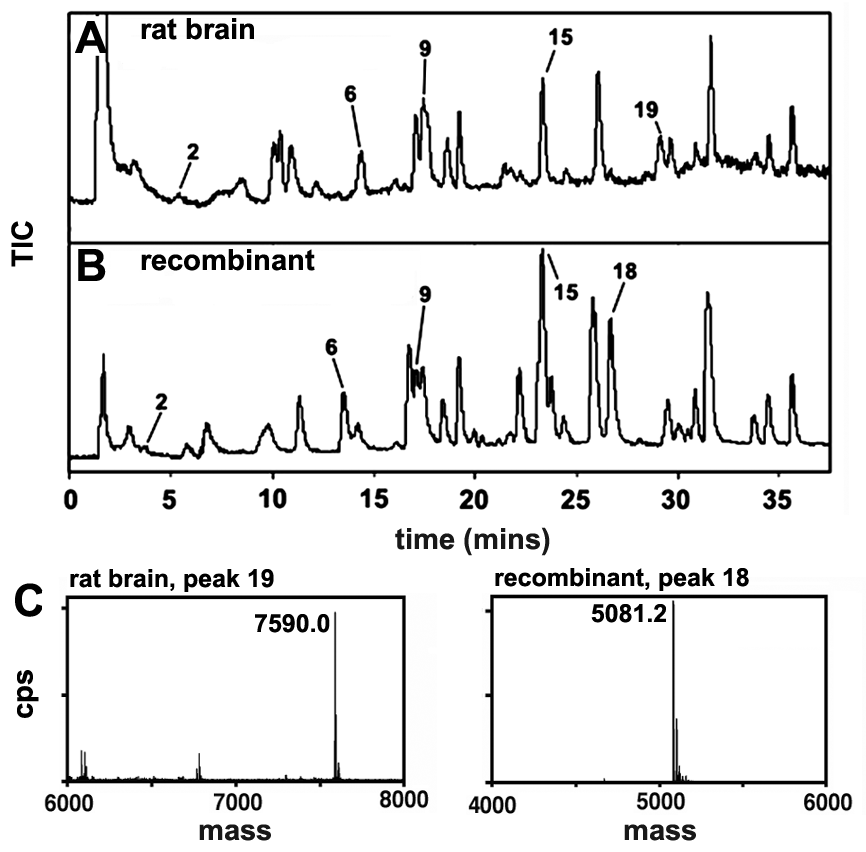

Supplement: Figure S2 — HnRNP A2 peptide mass spectra reveal dimethylation of Arg-254. (A & B) Proteins purified and digested with trypsin were separated and analyzed by LC-MS. Total ion current is plotted against the HPLC retention time. (C) Spectra of selected peaks that reveal a difference between recombinant and rat brain proteins. Only the latter generates a peptide of average mass 7590.0 Da (left panel, C), corresponding to residues 227–305 (peak 19 in A): the recombinant protein spectrum lacks this peak but has an additional peak containing a peptide with average mass 5081.2 Da (right panel in C) arising from residues 255–305 (peak 18 in B) as well as an additional mass of 2495.1 Da in peak 15 (corresponding to residues 227–254). (TIF) [file pone.0075669.s002.tif]

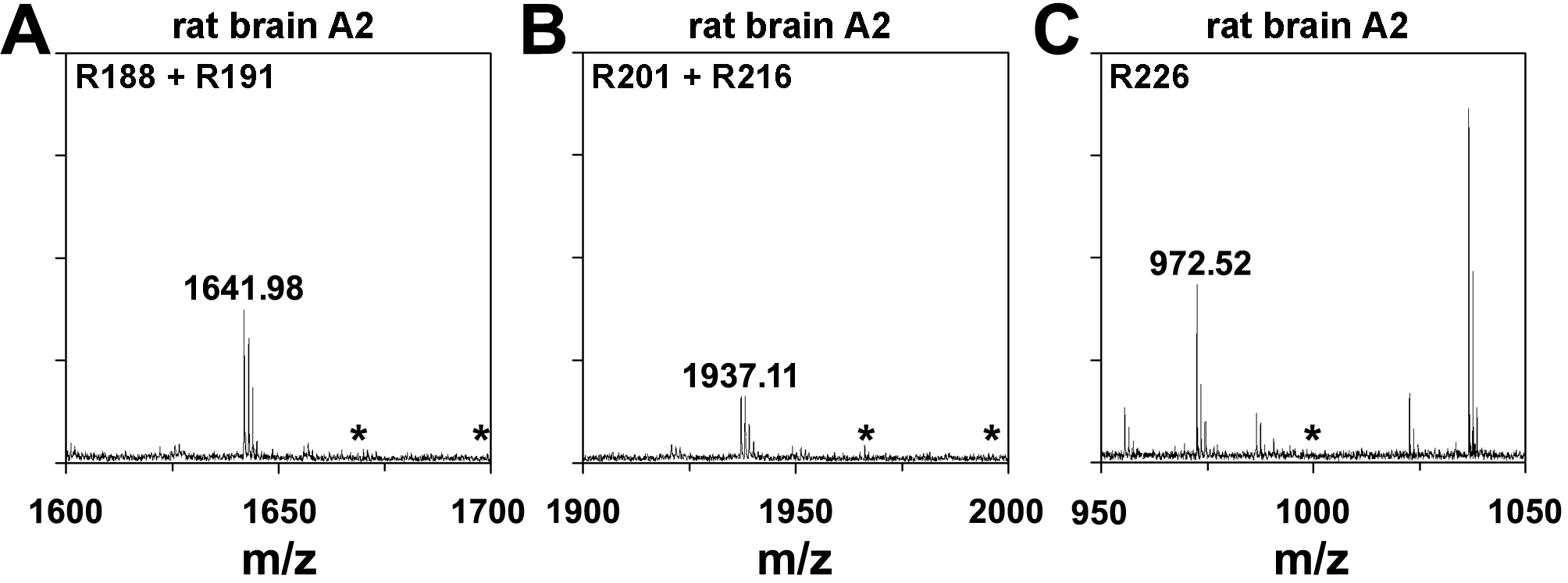

Supplement: Figure S3 — MALDI-TOF mass spectra of AspN peptides from rat brain hnRNP A2 confirm other RGG-like motifs are unmodified. The peaks arise from the peptides (A) 183–198 (containing Arg-188 & Arg-191), (B) 199–219 (containing Arg-201 & Arg-216) and (C) 220–229 (containing Arg-226). The asterisks mark the positions expected for dimethylation at each arginine residue (+28 or +56 Da). The minor peaks close to the right-hand asterisks in the left and center panels are outside the bounds of accuracy for +28 Da. (TIF) [file pone.0075669.s003.tif]

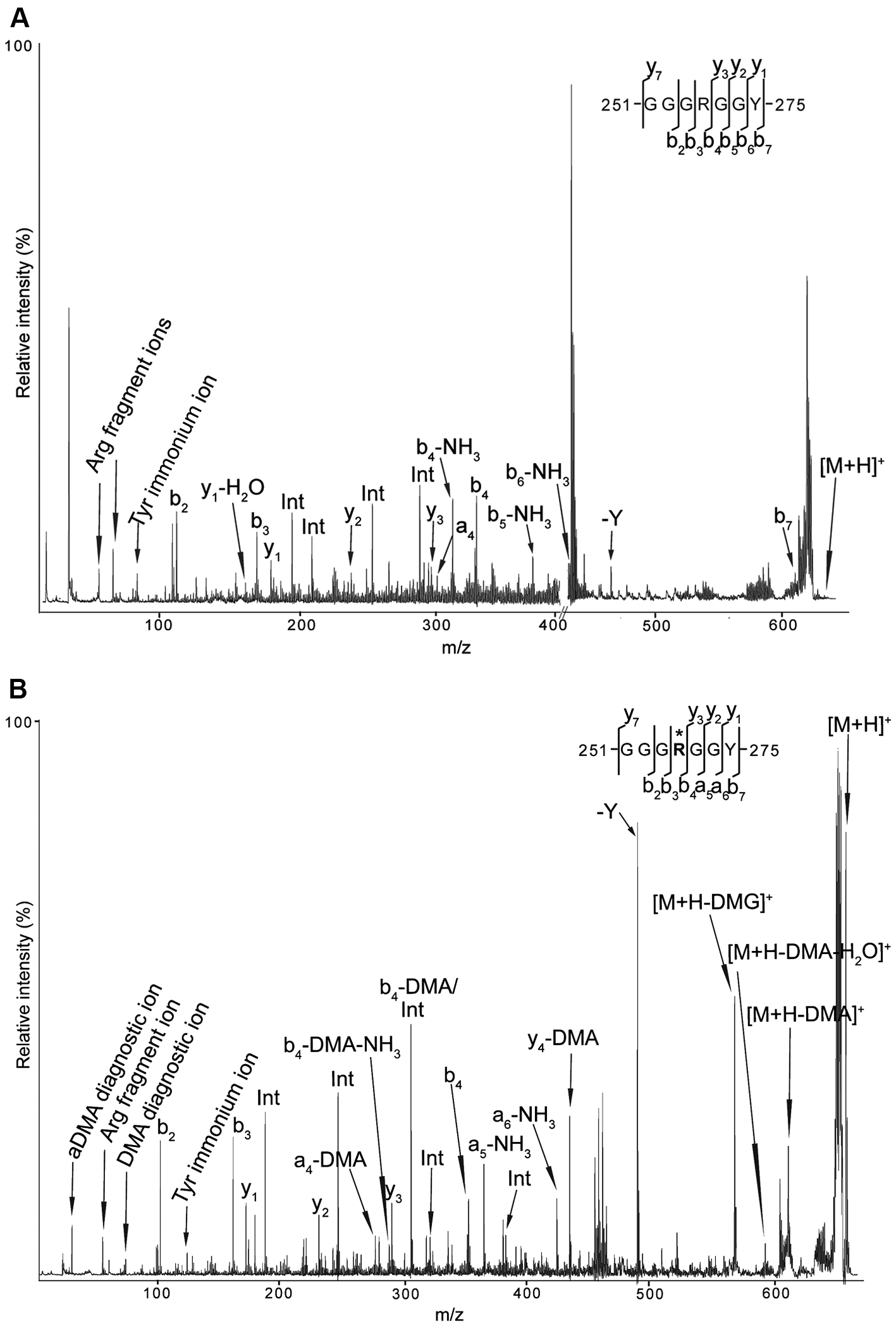

Supplement: Figure S4 — Diagnostic ions reveal asymmetric dimethylation of Arg-254. MALDI-TOF/TOF analysis of (A) m/z 623.2, corresponding to unmodified peptide 251-GGGRGGY-257 from chymotrypsin digest of recombinant A2 and (B) m/z 651.3 corresponding to peptide 251-GGGRGGY-257 with dimethylarginine modification from HPLC-purified rat brain hnRNP A2. Identified ions are indicated (refer to Fig. 2 of main text for additional details). DMG = dimethylated guanidinium, DMA = dimethylamine. (TIF) [file pone.0075669.s004.tif]

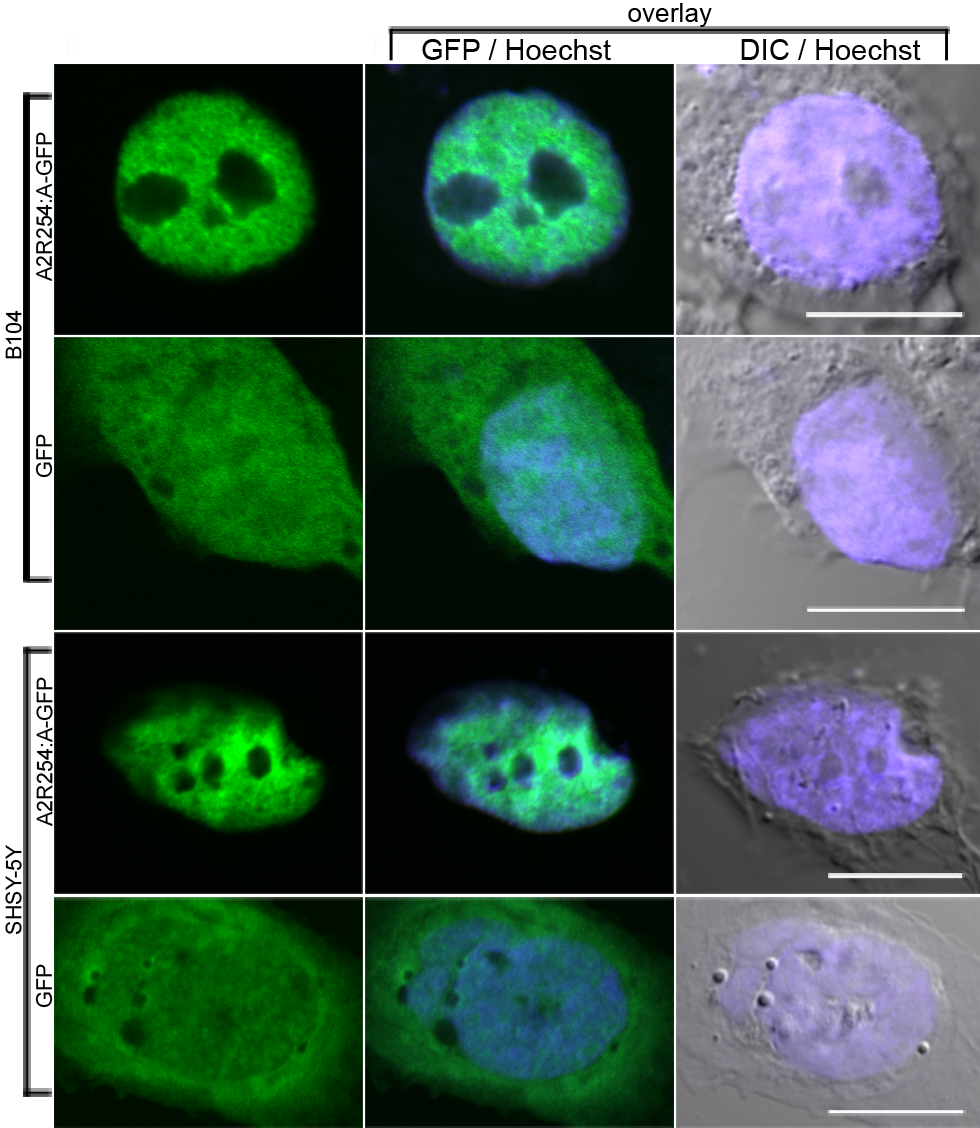

Supplement: Figure S5 — Transfected A2 point mutant in SH-SY5Y, B104. Transiently expressed A2R254A-GFP is exclusively localized to the nucleus of both B104 (upper panels) and SH-SY5Y transfected cell lines. Transfected GFP vector alone results in a diffuse nuclear and cytoplasmic signal. The nucleus is stained with Hoechst dye (blue) and is overlaid on the GFP signal (middle panels) and on corresponding DIC micrographs (right panels). Scale bar is 10 µm for B104 cells and 5 µm for SH-SY5Y cells. (TIF) [file pone.0075669.s005.tif]

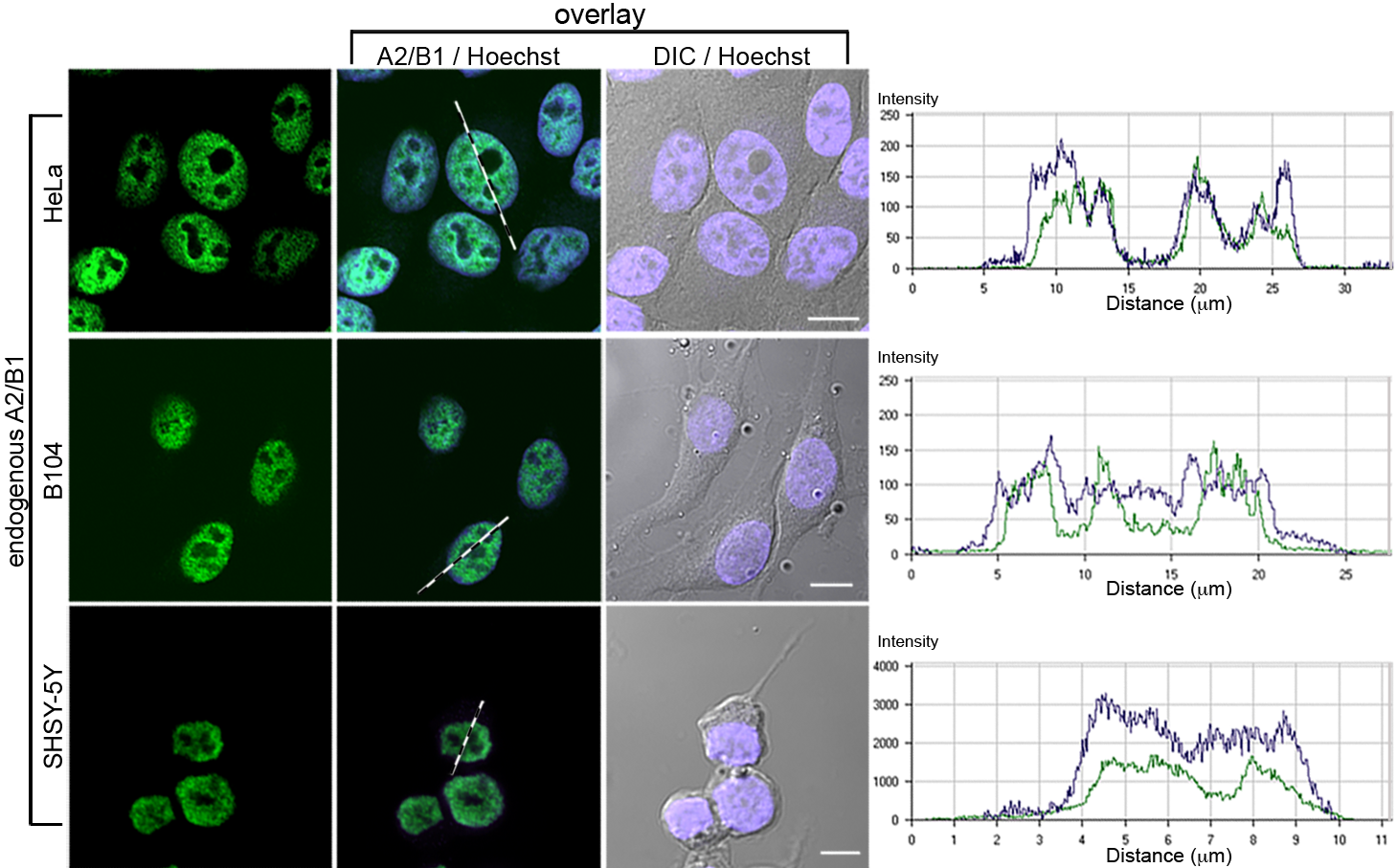

Supplement: Figure S6 — Field of view of endogenous A2 localized in the nucleus of HeLa, B104 and SH-SY5Y cells. Cells were fixed and immunostained using a rabbit polyclonal antibody against hnRNP A2/B1, followed by an anti-rabbit Alexafluor-488-congugated secondary antibody. The DNA was stained using Hoechst dye (blue) and the cells were imaged using confocal microscopy and DIC. The far right panel represents the profile of subcellular staining of overlaid A2/B1 / Hoechst examined using Zeiss LSM meta510 software. A dotted line for each cell type (A2/B1/Hoechst overlay panels) indicates the profile examined. Scale bar is 10 µm for HeLa and B104 cells and 5 µm for SH-SY5Y cells. (TIF) [file pone.0075669.s006.tif]
