# Supplementary material for: Arginine Methylation of hnRNP A2 Does Not Directly Govern Its Subcellular Localization
Source: PLoS One. 2013 Sep 30;8(9):e75669. doi: 10.1371/journal.pone.0075669 (PMC3787039; doi:10.1371/journal.pone.0075669)
Supplement: Table S1 — Selected mass spectrometry data for tryptic peptides of hnRNP A2 overexpressed in E. coli (recombinant) and isolated from rat brain. Mass spectrometry data for peaks from the LC chromatogram shown in Fig. S2A & S2B. Each peak confirms either the presence or absence of dimethylarginine for one of the five arginines highlighted for hnRNP B1 in yellow or magenta, respectively, in Fig. 7. (DOC) [file pone.0075669.s007.doc]

***Table S1****.* ***Selected mass spectrometry data for tryptic peptides of hnRNP A2 overexpressed in E. coli (recombinant) and isolated from rat braina***

| **Peak No.** | **Recombinant hnRNP A2** | | **Rat Brain hnRNP A2** | | **Theoretical** | **Residues** |
| --- | --- | --- | --- | --- | --- | --- |
|  | **RT (min)** | **Mass (Da)** | **RT (min)** | **Mass (Da)** | **Mass^b^** |  |
| 2 | 3.7 | 911.4 | 4.1 | 911.4 | 911.373 | 217-226 |
| 6 | 13.5 | 1012.5 | 14.0 | 1012.7 | 1012.436 | 192-201 |
| 9 | 17.1 | 1376.7 | 17.3 | 1376.7 | 1376.622 | 202-216 |
| 15a | 23.1 | 1187.7 | 23.3 | 1187.7 | 1187.640 | 126-135 |
| 15b | 23.1 | 2495.1 | *nd* | *nd* | 2494.032 | 227-254 |
| 18 | 26.7 | 5081.2 | *nd* | *nd* | 5081.030 | 255-305 |
| 19 | *nd* | *nd* | 29.3 | 7590.0 ^*^ | 7562.71 ^*^ | 227-305 |

*^a^* Mass spectrometry data for peaks from the LC chromatogram shown in Figure S2A & B. Each peak confirms either the presence or absence of dimethylarginine for one of the five arginines highlighted for hnRNP B1 in yellow or red, respectively, in Fig. 7.

*^b^*The theoretical mass refers to the protonated form. Not detected (*nd*)
