# Supplementary material for: Arginine Methylation of hnRNP A2 Does Not Directly Govern Its Subcellular Localization
Source: PLoS One. 2013 Sep 30;8(9):e75669. doi: 10.1371/journal.pone.0075669 (PMC3787039; doi:10.1371/journal.pone.0075669)
Supplement: Table S2 — Post-translational modification of hnRNP B1. (DOC) [file pone.0075669.s008.doc]

Table S2. Post-translational modification of hnRNP B1

| **m/z ^[charge]^** | **M*_r_*** | **Sequence*^a^*** | **Peptide*^b^* (enzyme*^c^*)** | **Arg** | **DMA** | **aDMA diagnostic ions*^d^*** | **Comments** |
| --- | --- | --- | --- | --- | --- | --- | --- |
| *hnRNP B1* |  |  |  |  |  |  |  |
| 535.8 ^[4+]^ | 2139.0 | RSQEMQEVQSSRSGRGGNF | 189-207 (c'trypsin) | 200,203 |  |  | ESI |
| 920.9 ^[2+]^ 614.3 ^[3+]^ | 1839.8 | GFGDSRGGGGNFGPGPGSNF | 208-227 (c'trypsin) | 213 |  |  | ESI |
| 555.6 ^[3+]^ | 1663.7 | RGGSDGYGSGRGFGDGY | 228-244 (c'trypsin) | 228,238 |  |  | ESI |
| 653.8 ^[2+]^ | 1305.5 | GSGRGFGDGYNGY | 235-247 (c'trypsin) | 238 |  |  | ESI |
| 935.4 ^[2+]^ 623.95 ^[3+]^ | 1868.8 | GGGPGGGNFGGSPGYGGGRGGY | 248-269 (c'trypsin) |  | 266 | yes | ESI, partial MMA and Arg detected in cultured cell lysates |

*^a^* residues with a modified mass are underlined

*^b^* note B1 contains an insert of 12 amino acids following residue 2of A2. The equivalent A2 sequence numbering can be obtained by subtracting 12 from each residue beyond Arg-15 of B1.

*^c^* c'trypsin=chymotrypsin

*^d^* peak at m/z 46 or neutral loss of 45 Da
